# Clinical Teaching Fellow Practice Within Complex Clinical–Educational Systems: An Activity Theory–Informed Case Study

**DOI:** 10.1111/tct.70534

**Published:** 2026-09-25

**Authors:** Nicola Jones, Zilley Khan, Kirsteen Watson

**Affiliations:** ^1^ Royal Papworth Hospital Cambridge UK; ^2^ University of Cambridge Cambridge UK

**Keywords:** activity theory, clinical learning environment, clinical teaching fellows, health professions education, Interprofessional education, medical education, workplace learning

## Abstract

**Background:**

Clinical teaching occurs where patient care and education coexist. Within the United Kingdom's National Health Service, workforce pressures constrain teaching and supervision. Clinical Teaching Fellow (CTF) roles have expanded in response but are often viewed as discrete posts rather than practices situated within complex clinical–educational systems. This study examined how tensions within and across these systems shaped CTF practice.

**Methods:**

This theory‐informed exploratory qualitative case study used third‐generation Activity Theory. All 10 members of a hospital medical education team were invited to a semistructured group interview; eight participated. An Activity Theory‐informed thematic approach was used to map the focal undergraduate clinical education activity system and examine tensions within it and at its intersections with adjacent systems.

**Findings:**

Participants included three CTFs, two education facilitators and three administrators. CTF practice involved teaching, programme coordination and administrative work within a multiprofessional team. Differing understandings of the activity's purpose and outcomes shaped practice. Professional language, continuity, experience and blurred divisions of labour influenced participation and work organisation. Relationships with clinical services, the medical school, postgraduate medical education and university‐based postgraduate study introduced competing demands. Participants negotiated tensions through communication, redistribution of responsibilities and closer working across professional roles.

**Conclusions:**

CTF roles are embedded within systems where educational, clinical and organisational priorities intersect. Third‐generation Activity Theory makes visible how relationships within and across these systems shape practice. Supporting CTF roles requires attention to how educational work is organised, coordinated and prioritised.

**Summary**

- Clinical Teaching Fellow (CTF) roles involve not only teaching but also coordinating and sustaining clinical placements within complex service environments.
- Tensions related to competing priorities, role boundaries and professional perspectives are routine and require ongoing, collaborative management.
- Effective support for CTF roles requires attention to how educational work is organised, coordinated and prioritised within clinical systems, not simply increasing teaching capacity.

## 1 Background

Clinical teaching in hospital settings brings together educators, clinicians, patients and learners within environments where care delivery and education must coexist [1, 2]. Teaching occurs alongside routine and unpredictable clinical work, requiring educators to balance planned learning with patient care and service delivery [3]. Differing priorities, temporal rhythms and organisational expectations therefore create tensions in how educational work is enacted [4].

Within the United Kingdom's National Health Service (NHS), staffing shortages, increasing demand and rota gaps constrain capacity for service delivery and education. The NHS Long Term Workforce Plan [5] aims to increase the number of healthcare professionals trained in the United Kingdom through university education and clinical placements, where learning occurs through teaching, supervision and participation in practice [6]. However, workforce pressures limit capacity for teaching and supervision [7], requiring continued negotiation between service and educational priorities.

Clinical educator roles have expanded in response [8], including Clinical Teaching Fellow (CTF) programmes, now common across NHS organisations [9, 10, 11]. CTFs are qualified doctors who support learners through teaching, placement coordination and engagement with clinical and educational teams [12]. Positioned at the intersection of clinical service, undergraduate education and professional development, they work across organisational contexts with differing priorities and expectations.

CTFs are qualified doctors who support learners through teaching, placement coordination and engagement with clinical and educational teams.

Despite the growing presence of CTF roles, the literature has largely focused on fellowship structures [13] and perceived benefits for learners [14], fellows [15] and faculty [16], with less attention to how CTF practice is organised within complex clinical–educational systems [17, 18]. CTF programmes are consequently conceptualised as discrete posts rather than situated forms of educational practice [4], limiting understanding of how they function within wider systems.

Examining CTF practice therefore requires attention to the wider systems in which this activity is organised and enacted. Activity Theory (AT) conceptualises work as collective, mediated activity shaped by relationships among participants, tools, rules, community and division of labour [19, 20, 21]. Its first generation focused on individual action mediated by cultural tools, the second on collective activity systems and the third on interactions between systems working towards shared or related purposes [19, 20]. Third‐generation AT is relevant because CTFs work across interacting clinical and educational systems. It makes visible how tensions arise within and between systems and shape collective, multiprofessional practice [21]. Such tensions are understood as inherent features of complex systems that may both constrain and drive change.

Accordingly, the aim of this study was to use third‐generation AT to examine how tensions within and between interacting activity systems shape CTF practice within a hospital medical education team and are negotiated in practice. It provides a theory‐informed, system‐oriented account of how clinical education is organised and might be supported beyond individual or programme‐level descriptions.

## 2 Methods

### 2.1 Study Design

We undertook a theory‐informed exploratory qualitative case study of CTF practice, using third‐generation AT to orient data collection and analysis. The framework enabled examination of how activity was mediated within the focal undergraduate clinical education system and how tensions arose within it and at its intersections with adjacent clinical, educational and organisational systems.

### 2.2 Case Setting

The study was situated within the CTF programme of a specialist NHS hospital. The programme supports a cohort of CTFs each year, including both full‐time education posts and combined clinical–educational roles. CTFs work within a multiprofessional medical education team that includes education facilitators from nursing, physiotherapy, and healthcare science, alongside administrative staff.

The team supports undergraduate medical students through ward‐based learning, small‐group teaching, practical skills training, simulation and placement coordination. CTFs also teach postgraduate and locally employed doctors, complete a postgraduate certificate in medical education and undertake an educational project. The programme was selected as an instrumental case in which multiple clinical and educational systems intersected.

### 2.3 Participants and Recruitment

All 10 medical education team members were invited following introduction of the study at a routine team meeting and an email outlining its aims, voluntary participation and confidentiality. Eight participated: three CTFs, two education facilitators and three administrative staff, representing different backgrounds, working patterns and levels of experience (Table 1). One CTF and the Medical Education Training Programme Director were unable to attend because of planned leave and sickness absence, respectively.

**TABLE 1:** Broad characteristics of participants in the semistructured group interview, presented by role within the medical education team.

Role in medical education team	Number	Professional background	Working pattern	Experience in team
Clinical Teaching Fellow	3	Medicine	Full‐time	Less than 1–1 year
Education facilitator	2	Nursing; physiotherapy	Part‐time	2 and 10 years
Administrator	3	Administration	Full‐time	1, 2 and 10 years

### 2.4 Data Collection

Data were generated through a 90‐min semistructured group interview. The group format enabled examination of collective activity and allowed participants to build on and challenge accounts of shared practice. Broad questions‐oriented discussion through the activity‐system lens while allowing further exploration of participants' responses.

The interview was conducted by the lead researcher. Participants were introduced to the concept of an activity system and invited to describe their work in relation to its purpose, tools, rules, community and division of labour. They also identified interacting systems that shaped or intersected with their work, including clinical services, the medical school and university‐based postgraduate education.

As discussion progressed, the activity system was co‐constructed by the group. Points of uncertainty or divergence were explored further, including whether these reflected tensions within or between elements of the activity system and how such tensions were negotiated. The lead researcher facilitated equitable participation and remained attentive to potential hierarchical influences. The session was audio‐recorded and transcribed verbatim. Transcripts were pseudonymised and stored securely. The full interview schedule is provided in Supplementary File Data S1.

### 2.5 Data Analysis

Data were analysed using an AT‐informed thematic approach. The co‐constructed activity system provided the starting point for mapping participants' accounts. The transcript was coded in relation to the subject, object, tools, rules, community, division of labour and intended outcomes, while remaining open to perspectives and relationships not captured by these components.

Analysis examined recurrent patterns in the purpose, participation, professional relationships and organisation of activity. Accounts of difficulty, misalignment or strain were considered in relation to components of the focal system and its intersections with adjacent systems. Recurring misalignments shaping work were interpreted as tensions, with attention to how they were experienced and negotiated. The lead researcher conducted the primary analysis, developing interpretations iteratively with co‐authors.

### 2.6 Reflexivity

The lead researcher held a senior role within the medical education team at the study site and had previously contributed to the development of the CTF programme. This insider position afforded contextual understanding but also introduced potential for assumptions and existing relationships to influence data generation and interpretation.

Reflexive strategies included maintaining a reflective record, distinguishing participants' accounts from the researcher's experience and attending to power dynamics. Participants refined or challenged the emerging representation during the interview and were invited to comment on the preliminary descriptive account.

Interpretations were discussed with co‐authors with expertise in AT and CTF programmes. These discussions served as analytic checks rather than validation exercises, supporting theoretical and contextual grounding.

### 2.7 Ethics

The study was conducted in accordance with the Declaration of Helsinki. Ethical approval was granted by the Cambridge Psychology Research Ethics Committee on 19 July 2022 (Application No. PRE.2022.055). Written informed consent was obtained from all participants, and all data were pseudonymised.

## 3 Findings

The findings are presented as a narrative account of how participants understood and organised the team's educational activity, the tensions arising within the focal activity system and at its intersections with adjacent systems, and how these were negotiated in practice.

### 3.1 Constructing and Maintaining the Purpose of Educational Activity

For the purposes of this study, CTFs were treated as the focal subject of the activity system. Participants initially described medical students as the focus of the team's activity and its intended outcome as enabling their ‘education and progression’. In the analytical mapping, the object was formulated as delivering undergraduate clinical education through placements, directed towards students developing the knowledge, skills and professional capabilities required for progression to practice.

Participants described this as collective activity rather than the work of CTFs alone. It was enacted through ward‐based learning, structured small‐group teaching, practical skills training, simulation and placement coordination. Teaching materials, practical skills equipment, simulation resources and digital platforms mediated this activity, but their use depended on collaboration between CTFs, education facilitators, administrators and clinicians.

**FIGURE 1:**
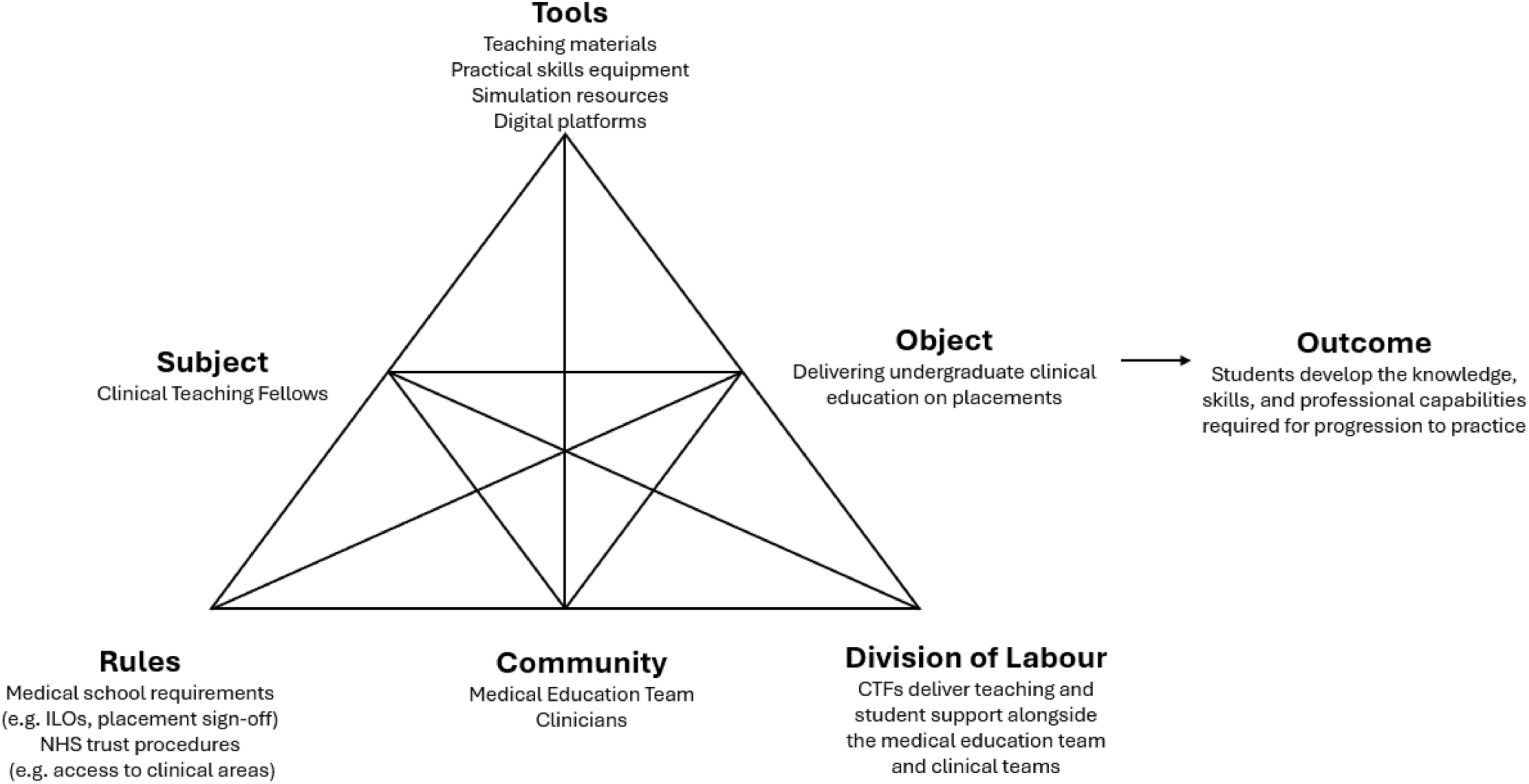
Activity system representing Clinical Teaching Fellow (CTF) practice within undergraduate medical education. The figure illustrates the elements of the activity system, including subject (CTFs), object (undergraduate clinical education), mediating tools, rules, community and division of labour, and their relationship to intended outcomes.

Medical‐school requirements—including the curriculum, intended learning outcomes, assessment processes and placement sign‐off—provided rules within which the team worked. Funding arrangements further bounded the remit of activity, whereas hospital procedures, workforce availability and access to clinical areas shaped what could be delivered locally. Figure 1 brings these elements together in the focal undergraduate clinical education activity system constructed from participants' accounts.

Although participants shared this immediate educational purpose, their accounts revealed different orientations towards its wider meaning. CTFs, all of whom had worked within the team for 1 year or less, described how the immediate demands of programme delivery could displace its broader purpose. One explained: ‘Day to day I am not thinking this person needs to be a doctor; I am thinking we have these students for a week, we must do these things for them … a bit like a conveyor belt’ (CTF). For some participants, concentrating on immediate tasks made a complex programme manageable. For others, it reduced their sense of connection to the collective purpose. An administrator described having insufficient ‘headspace to think about the bigger picture’, as work became ‘a snowball of day‐to‐day monotonous tasks’ (administrator).

In contrast, an education facilitator located the team's activity within a broader outcome extending from medical education to workforce experience and patient care: ‘If we take a step back, our outcome is patient care … improving doctors' experience and longevity … bolster teaching for medical students and then, in turn, patient care’ (education facilitator). Immediate programme delivery and this broader educational purpose therefore coexisted but were unevenly foregrounded in practice.

Funding arrangements also influenced the boundary between undergraduate and postgraduate education. One CTF explained that medical students were prioritised because of ‘our funding stream’, meaning that engagement with postgraduate doctors was often directed towards supporting their contribution to undergraduate teaching. Another considered it ‘a shame to only help postgraduates as we want them to do things for the students’, adding that ‘if we have time then we should teach them’ (CTFs). These accounts revealed differing views about whether postgraduate education should be valued principally for its contribution to undergraduate teaching or as an outcome in its own right.

**FIGURE 2:**
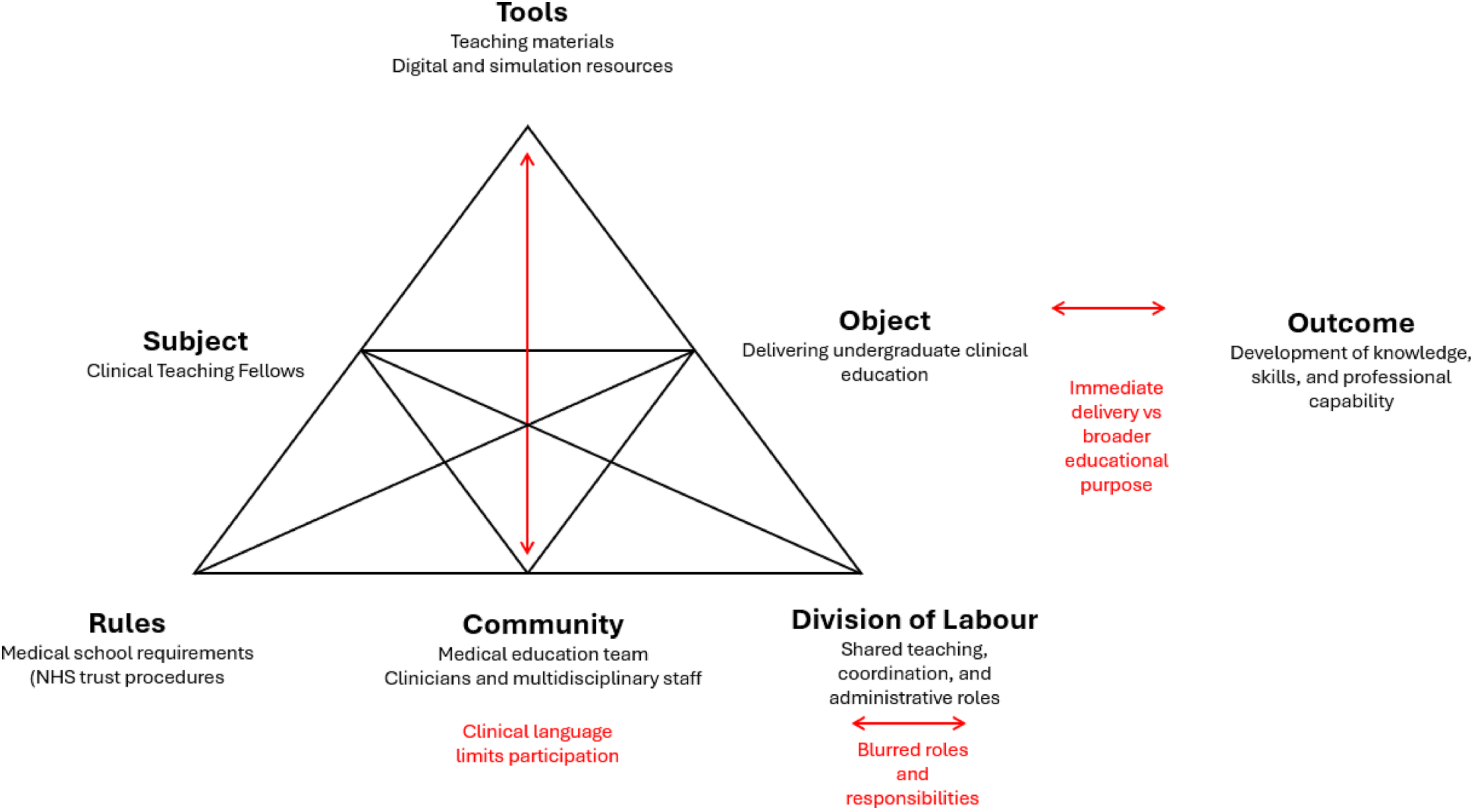
Tensions within the activity system supporting undergraduate medical education. This figure illustrates tensions within key elements of the activity system, including object orientation (immediate delivery versus broader educational purpose), communication within the multiprofessional community (clinical language shaping participation) and division of labour (blurred roles and responsibilities). Tensions are represented by bidirectional arrows indicating areas of misalignment within the system.

Figure 2 presents the same focal undergraduate clinical education activity system as Figure 1, with the principal tensions identified through analysis superimposed.

### 3.2 Organising Collective Work Across Professional Boundaries

Participants situated CTF practice within a wider multiprofessional community. In addition to the immediate medical education team, this included resident doctors, consultants, multidisciplinary staff, Information Technology and Human Resources, the affiliated medical school and other placement providers. Participants emphasised the multivoiced nature of this work, explaining that ‘each of us brings something different to the team’. These different forms of expertise supported educational delivery but also shaped who could participate in programme planning and how responsibilities were distributed.

Tensions were evident within the community, particularly in relation to differences in professional background and ways of working. Planning and coordination involved clinical and administrative staff with differing expertise. Language was one way in which these differences affected participation. An administrator described how clinically oriented discussions could limit administrative involvement: ‘There is a language barrier … we don't understand, so you have those discussions, then just tell us what you need us to do’ (administrator). An education facilitator clarified that the language used in programme discussions had become ‘more clinically based’ (education facilitator). Clinical language therefore mediated communication for some participants while simultaneously creating a barrier for others.

Similar tensions were evident in the division of labour. Although formal roles distinguished teaching, facilitation, coordination and administration, participants described responsibilities as unclear and increasingly blurred. As CTFs assumed responsibility for developing and coordinating new programmes, administrative staff sometimes experienced reduced involvement. One administrator explained that ‘the CTF did everything, from start to finish … I had very minimal control over anything or involvement’ (administrator). A CTF explained that this had not been deliberate: Although developing a new programme, determining and communicating what administrative support was required could itself be difficult.

An education facilitator linked this difficulty to clinicians' previous ways of working. Clinicians were accustomed to collaborating within clinical teams but often had limited experience of working directly with administrators or delegating programme‐related tasks. Incoming CTFs therefore needed to understand the expertise within the administrative team and what work could appropriately be shared. Without this understanding, programme knowledge risked remaining with annually rotating CTFs or within documentation rather than becoming shared institutional knowledge.

Differences in continuity and tenure also shaped participation. Longer standing members held tacit knowledge of programmes, whereas newer or rotating CTFs required time to become familiar with expectations, influencing engagement in planning and coordination. An administrator with 10 years' experience described how continuity had supported confidence and autonomy: ‘Over the last few years I have not needed so much support. I felt as though I could run with the programme … it's my baby and I am running with this’ (administrator). However, experience was not the only influence on ownership. An administrator with less time in the team also sought greater responsibility, explaining that repeatedly undertaking the same tasks would diminish their enjoyment of a role they otherwise found ‘really rewarding’ (administrator). Participation was therefore shaped by the interaction between experience, professional background, opportunities for involvement and individual aspirations, rather than tenure alone.

Participants also described differences in how work was prioritised, with teaching activity often foregrounded and administrative tasks addressed later. These patterns were attributed to programme complexity, expanding student numbers and staffing changes.

Illustrative examples are presented in Table 2, which provides excerpts demonstrating how these tensions were expressed in practice.

**TABLE 2:** Illustrative quotations showing tensions within the focal undergraduate clinical education activity system and at its intersections with adjacent systems. Quotations are attributed by participant group to situate the differing professional perspectives represented in the analysis.

Activity‐system focus	Analytic description of tension	Participant group	Illustrative quotation
Object and outcome of activity	Immediate programme delivery displacing attention from the wider purpose of undergraduate education	CTF	‘Day to day I am not thinking this person needs to be a doctor; I am thinking we have these students for a week, we must do these things for them … a bit like a conveyor belt’.
	Broader outcome‐oriented framing extending from undergraduate education to workforce experience and patient care	Education facilitator	‘If we take a step back, our outcome is patient care … improving doctors' experience and longevity … bolster teaching for medical students and then, in turn, patient care’.
Community and mediating tools	Clinically oriented language limiting administrative participation in multiprofessional programme planning	Administrator	‘There is a language barrier … we do not understand, so you have those discussions, then just tell us what you need us to do’.
	Limited experience of working across clinical and administrative professional boundaries	Education facilitator	‘As a clinician, we are good at working with other people on the ward, but we have never really worked with administrators … the fellows coming in need to learn what the admin team can do and help with’.
Division of labour	Increasing CTF involvement in programme development reducing administrative involvement and ownership	Administrator	‘The CTF did everything, from start to finish … I had very minimal control over anything or involvement’.
	Continuity and experience shaping confidence, autonomy and programme ownership	Administrator	‘Over the last few years I have not needed so much support. I felt as though I could run with the programme … it's my baby and I am running with this’.
Interacting systems	Undergraduate funding requirements shaping the purpose of engagement with postgraduate doctors	CTF	‘Our role in postgraduate is solely with the purpose of engaging them to help teach the students’.
	Competing commitments across clinical and educational roles	Education facilitator	‘I am doing two separate jobs. I cannot fully commit to one particular role or another … pulled in two different directions’.

### 3.3 Tensions Across Interacting Systems

**FIGURE 3:**
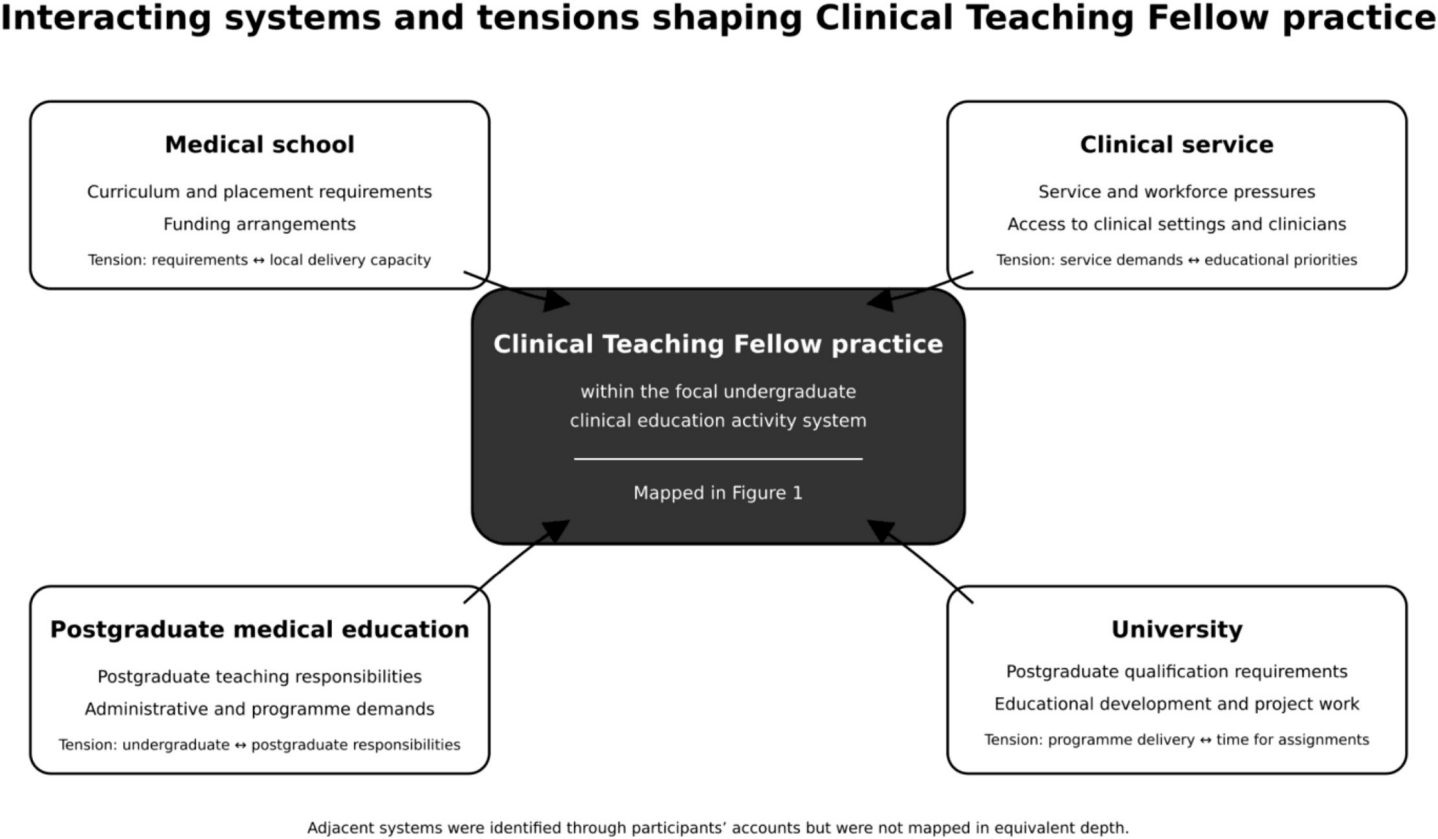
Interacting systems and tensions shaping Clinical Teaching Fellow practice. Participants described CTF practice as situated in relation to medical‐school, clinical‐service, postgraduate medical education and university systems. Arrows indicate requirements and demands acting on the focal undergraduate clinical education activity system; labels identify tensions arising between differing priorities.

Participants described CTF practice as situated in relation to four adjacent systems: the medical school, clinical service, postgraduate medical education and the university through which CTFs undertook postgraduate qualifications (Figure 3). The focal undergraduate clinical education activity system was mapped in detail, whereas adjacent systems were identified through participants' accounts of the demands, requirements and relationships that shaped their work but were not mapped in equivalent depth.

The medical‐school system shaped activity through curriculum and placement requirements and through the funding arrangements defining the team's undergraduate remit. Participants' accounts indicated a tension between these external requirements and the team's local capacity to deliver them, particularly amid expanding student numbers, staffing changes and workforce constraints.

Clinical services shaped CTF practice through service pressures, clinician availability and access to clinical settings in which learning occurred. All participating CTFs held full‐time education posts and therefore did not describe personally balancing concurrent clinical and educational employment. Direct accounts of navigating clinical and educational roles came from education facilitators working part‐time in education alongside clinical practice. One described ‘doing two separate jobs’ and being ‘pulled in two different directions’ because they could not fully commit to either role (education facilitator). Another explained: ‘I am one person, but I am in this activity system and almost another activity system … sometimes these systems have different outcomes’ (education facilitator). These accounts situated CTF practice within a wider team whose collective activity was shaped by tensions between clinical service demands and educational priorities.

Postgraduate medical education introduced additional teaching, administrative and programme responsibilities. Participants with responsibilities across undergraduate and postgraduate education described having to prioritise one area over another, with concern that other work might consequently be delayed or missed. The tension therefore concerned not only clinical service and education but also competing responsibilities across undergraduate and postgraduate educational systems.

The university introduced requirements associated with CTFs' postgraduate qualifications, including time needed to complete assignments and project work. Participants described a tension between maintaining programme delivery and protecting sufficient time for postgraduate study. This situated CTF professional development within the same network of competing demands rather than as an activity separate from their educational practice.

### 3.4 Negotiating Tensions in Practice

Participants described negotiating tensions through pragmatic practices aimed at sustaining educational activity.

Tensions across systems were managed through prioritisation and renegotiation of tasks, supported by communication. An administrator described explaining to a colleague that another matter had to be prioritised and that their request would be revisited the following week (administrator). Participants also sought to reconnect day‐to‐day work with its broader educational purpose. Multiprofessional teaching sessions were described as helping team members ‘see the bigger picture’ (education facilitator).

Adjustments to the division of labour included assigning administrative leads for year groups and pairing incoming CTFs with experienced administrators, supporting coordination. A newly appointed CTF described the proposed partnership between CTFs and administrators as the ‘perfect marriage’ (CTF), reflecting the potential value of combining CTFs' educational contribution with administrators' continuity and programme knowledge.

Across these examples, participants emphasised collaboration and informal negotiation as key mechanisms for managing tensions. These responses represented local adaptations that allowed educational activity to continue, including clearer communication, greater administrative ownership and closer working between incoming CTFs and established team members. Participants described adjustments to existing working practices rather than wider changes to the objects, roles or relationships across the interacting systems.

Across these examples, participants emphasised collaboration and informal negotiation as key mechanisms for managing tensions.

## 4 Discussion

This theory‐informed case study conceptualised CTF practice as collective, mediated activity situated within interacting systems. It responds to calls to move beyond individualised accounts of teaching roles and attend to the organisational and contextual conditions shaping educational practice [2, 4].

Participants described undergraduate clinical education shaped by tools, rules and relationships within a multiprofessional community and by the priorities of the medical school, clinical service, postgraduate medical education and university. Viewed through AT, CTF practice was organised through collective coordination within historically and institutionally situated systems, rather than individual teaching actions alone [19, 20].

A central contribution is the analysis of tensions within the focal activity system and at its intersections with adjacent systems. Although participants shared an orientation towards undergraduate education, they foregrounded different outcomes: immediate delivery, learners' longer term development or an indirect contribution to patient care. In AT terms, these represent different orientations towards the object and outcomes of collective activity rather than individual inconsistency [22].

Differences in professional background and team tenure influenced participation in planning, coordination and decision‐making. Clinical terminology facilitated communication for some while limiting others' involvement. Continuity supported access to tacit programme knowledge, although aspirations for autonomy and ownership were not determined by tenure alone. These dynamics shaped whose perspectives were foregrounded, consistent with accounts of tacit and situated professional learning [3, 23].

Despite formal roles, responsibilities for teaching, coordination and administration were frequently negotiated. CTFs assumed substantial organisational responsibility during programme development, whereas administrative involvement and ownership varied. This reflected programme expansion, workforce pressures, turnover and limited experience of working across clinical–administrative boundaries. From an AT perspective, division of labour was produced dynamically through everyday work rather than determined by role descriptions alone [4, 20].

Boundary tensions arose as medical‐school requirements were reconciled with local capacity, service pressures affected access to clinicians and learning environments, undergraduate and postgraduate education generated competing responsibilities, and university requirements competed with programme delivery. Their expression varied by role: education facilitators described moving between clinical and educational work, whereas CTFs emphasised programme delivery, postgraduate responsibilities and university assignments. CTF practice was therefore situated within systems operating with different priorities, expectations and temporal demands, made visible through multiprofessional perspectives [20].

CTF practice was therefore situated within systems operating with different priorities, expectations and temporal demands, made visible through multiprofessional perspectives.

Participants described negotiating tensions through pragmatic, stabilising adaptations, including communication about priorities, redistribution of responsibilities and adjustments to team working. Administrative leads and partnerships between incoming CTFs and experienced administrators supported continuity and coordination. Although these adaptations sustained activity, their reliance on local relationships and informal negotiation made them vulnerable to changes in personnel, workload or programme requirements. Blurred boundaries could produce duplication or uncertainty, whereas competing priorities could constrain teaching opportunities during periods of service pressure. Dependence on individual adaptability may therefore limit the sustainability and scalability of educational provision.

Dependence on individual adaptability may therefore limit the sustainability and scalability of educational provision.

These findings align with descriptions of clinical educators balancing patient care, service delivery and education within complex workplaces [1, 2, 3, 4]. Competing priorities, negotiated boundaries and coordination challenges occur across clinical educator roles. Our analysis extends this literature by using AT to understand them as features of collective activity and interacting systems rather than individual roles or behaviours. It positions CTF roles as practices embedded within systems where educational, clinical and institutional priorities intersect, rather than simply as a means of increasing teaching capacity. AT made visible how purpose was interpreted, participation mediated, responsibilities negotiated and external requirements translated into local delivery. Attention to objects, community, division of labour and cross‐system priorities may support more sustainable role development [4]. Beyond its analytic value, AT may therefore provide a practical means of examining clinical education, shifting attention from tensions as individual operational problems towards the organisational arrangements through which they are produced and negotiated.

### 4.1 Limitations and Future Directions

This single, theory‐informed case study sought analytic transferability rather than statistical generalisation. The group interview enabled exploration of collective activity and multiple professional perspectives, although existing relationships may have influenced participation. The focal system was examined more deeply than adjacent systems, and the cross‐sectional design captured practice at one point in time. Future research could examine whether comparable tensions arise across different institutional contexts, include perspectives from medical schools, clinical services, postgraduate medical education and university providers and examine longitudinally how changes to programme structures, professional relationships or divisions of labour shape practice. Comparative multisite research could also explore which organisational conditions support sustainable CTF programmes across different settings.

### 4.2 Implications

CTF roles should be designed with attention to the activity systems in which they are embedded. Framing CTFs primarily as additional teaching capacity risks overlooking the conditions shaping practice. Organisations should clarify the relationship between undergraduate education and service priorities, establish shared forums and language for multiprofessional planning and recognise the coordination and administrative labour sustaining placements.

Structured handover between rotating CTFs and established staff, clarification of CTF and administrative responsibilities and protected postgraduate study time may reduce reliance on informal negotiation. Mechanisms for reconciling medical‐school requirements with local capacity may further support sustainability. By making focal and adjacent systems explicit, AT offers organisations a practical framework for developing shared understanding and identifying tensions within and between clinical education systems [24].

## 5 Conclusion

Third‐generation AT conceptualised CTF practice as collective, system‐embedded activity shaped by relationships with medical‐school, clinical‐service, postgraduate education and university systems. CTF roles therefore represent more than additional teaching capacity: They are educational practices situated within clinical, educational and organisational relationships. Supporting them requires attention to how educational work is organised, coordinated and prioritised. Multisite and longitudinal research should examine how these relationships and tensions vary across settings and respond to organisational change.

CTF roles therefore represent more than additional teaching capacity: They are educational practices situated within clinical, educational and organisational relationships.

## Author Contributions

**Nicola Jones:** conceptualization, methodology, investigation, data curation, formal analysis, project administration, and writing – original draft. **Zilley Khan:** methodology, formal analysis, validation, supervision, and writing – review and editing. **Kirsteen Watson:** methodology, formal analysis, validation, supervision, and writing – review and editing. All authors approved the final manuscript and agree to be accountable for all aspects of the work.

## Funding

The authors have nothing to report.

## Ethics Statement

The study was conducted in accordance with the Declaration of Helsinki. Ethical approval was granted by the Cambridge Psychology Research Ethics Committee. Written informed consent was obtained from all participants.

## Consent

The authors have nothing to report.

## Conflicts of Interest

The authors declare no conflicts of interest.

## Supporting information


**Data S1:** Supporting information.

## Data Availability

Data are available from the corresponding author on reasonable request.
