# Supplementary material for: Clinical Teaching Fellow Practice Within Complex Clinical–Educational Systems: An Activity Theory–Informed Case Study
Source: Clin Teach. 2026 Sep 25;23(6):e70534. doi: 10.1111/tct.70534 (PMC13613996; doi:10.1111/tct.70534)
Supplement: Supplementary file 1 [file TCT-23-e70534-s001.docx]

**Semi-structured group interview schedule**

**Purpose, object and outcome of activity**

- Can you describe the work or activity of the team?
- What is the main purpose of this activity?
- What is the team seeking to achieve or change through its work?
- Who benefits from, or is affected by, the activity?
- What would a successful outcome look like?
- How do you know whether the activity is effective?

**Organisation of the activity system**

- Can you describe the workplace or system within which this activity takes place?
- Who participates in the activity, both within and beyond the immediate team?
- How are tasks and responsibilities divided among those involved?
- What tools or resources support the activity?
- What formal or informal rules, expectations or constraints shape the work?
- What other people, teams, systems or organisations interact with or influence the activity?

**Tensions, development and change**

- What difficulties, tensions or competing demands arise in undertaking this work?
- Where do these tensions arise—for example, within the team or between different teams, organisations or systems?
- How have these tensions developed over time?
- Have they led to changes in how the team works?
- How does the team currently respond to or negotiate these tensions?
- How might the activity be organised more effectively in the future?
- What changes, resources or forms of support would enable this?
